# Consequences of BMPR2 Deficiency in the Pulmonary Vasculature and Beyond: Contributions to Pulmonary Arterial Hypertension

**DOI:** 10.3390/ijms19092499

**Published:** 2018-08-24

**Authors:** Adam Andruska, Edda Spiekerkoetter

**Affiliations:** 1 Department of Medicine, Division of Pulmonary and Critical Care, Stanford University, Stanford, CA 94305, USA; aandrusk@stanford.edu; 2 Wall Center for Pulmonary Vascular Disease, Stanford University, Stanford, CA 94305, USA; 3 Cardiovascular Institute, Stanford University, Stanford, CA 94305, USA

**Keywords:** pulmonary hypertension, Bone Morphogenetic Protein Receptor II, vascular disease, genetic predisposition to disease

## Abstract

Since its association with familial pulmonary arterial hypertension (PAH) in 2000, Bone Morphogenetic Protein Receptor II (BMPR2) and its related signaling pathway have become recognized as a key regulator of pulmonary vascular homeostasis. Herein, we define BMPR2 deficiency as either an inactivation of the receptor, decreased receptor expression, or an impairment of the receptor’s downstream signaling pathway. Although traditionally the phenotypic consequences of BMPR2 deficiency in PAH have been thought to be limited to the pulmonary vasculature, there is evidence that abnormalities in BMPR2 signaling may have consequences in many other organ systems and cellular compartments. Revisiting how BMPR2 functions throughout health and disease in cells and organs beyond the lung vasculature may provide insight into the contribution of these organ systems to PAH pathogenesis as well as the potential systemic manifestation of PAH. Here we review our knowledge of the consequences of BMPR2 deficiency across multiple organ systems.

## 1. Introduction

Nearly 20 years ago, genetic linkage studies connected Bone Morphogenetic Protein Receptor II (BMPR2) to the development of familial pulmonary arterial hypertension (PAH) [1,2]. In the wake of the research that followed, the role of BMPR2 expanded beyond its origins in bone mineralization [3] to that of being a central key mediator of not only development but vascular homeostasis [4,5]. Beyond vascular biology, BMPR2 is necessary for development, organogenesis, and cancer pathogenesis [6,7]. Despite this, it remains strongly associated with its role in PAH pathogenesis, with an estimated 80% of familial and 20% of idiopathic PAH patients carrying a heterozygous BMPR2 mutation [8,9]. As we will detail below, BMPR2 mutations in PAH confer a more severe clinical phenotype. While BMPR2 signaling is often reduced in patients without a receptor mutation, a large percentage of patients with mutations never develop disease. Although systemic manifestations of PAH are increasingly recognized, the dramatic plexiform vascular lesions of PAH are confined to the lungs. Therefore, better understanding of BMPR2’s function in and out of the lungs may allow us to explain these paradoxes.

**Figure 1:**
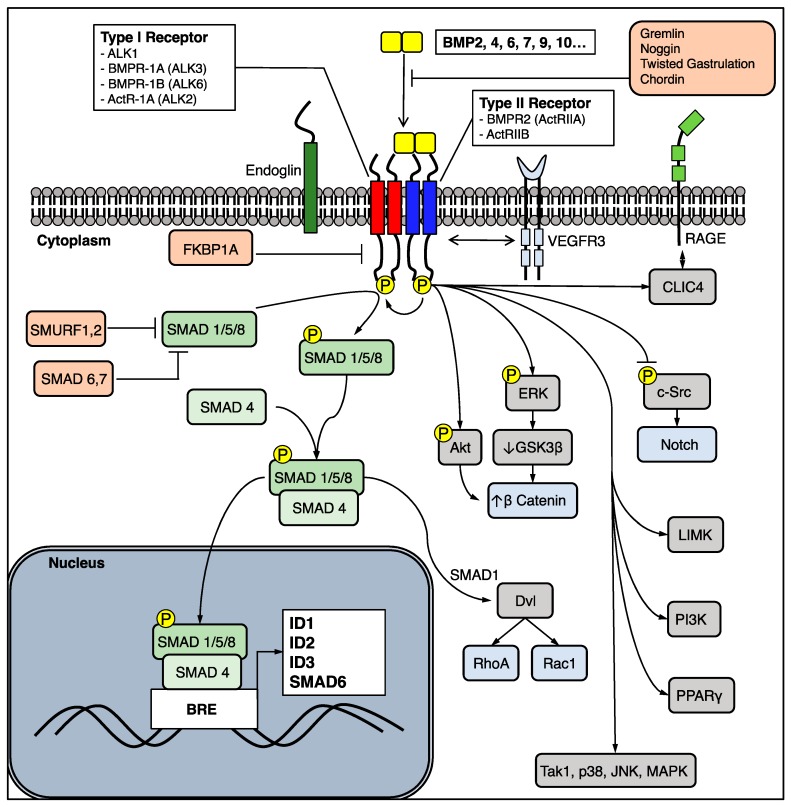
A schematic representation of the BMPR2 signaling pathway illustrating the potential canonical (green) and noncanonical (grey, blue) targets that can be activated. Note that downstream signaling targets are influenced by the combination of type 1 and type 2 receptors as well as the ligand. ActR = activin Receptor; Akt = protein kinase b; ALK = activin-like receptor; BMP = bone morphogenetic protein; BMPR = Bone Morphogenetic Protein Receptor; BRE = BMP response element; c-Src = proto-oncogene tyrosine-protein kinase Src; CLIC4 = chloride intracellular channel 4; Dvl = Dishevelled; Erk = extracellular signal-regulated kinase; FKBP1A = FK binding protein 1A; GSK3-β = glycogen synthase kinase 3-β; ID = Inhibitor of differentiation; JNK = c-Jun N-terminal kinase; LIMK = Lin11, Isl-1, and Mec-3 domain kinase; MAPK = Mitogen-activated protein kinase; PI3K = Phosphoinositide 3-kinase; PPARγ = peroxisome proliferator-activated receptor gamma; Rac1 = Ras-related C3 botulinum toxin substrate 1; RAGE = receptor for advanced glycation end products; RhoA = Ras homolog gene family, member A; SMAD = Mothers against decapentaplegic; SMURF = SMAD-specific E3 ubiquitin protein ligase; Tak1 = Transforming growth factor-β activated kinase 1; VEGFR3 = Vascular endothelial growth factor receptor 3.

BMPR2 is a “type 2” member of the transforming growth factor (TGF)-β superfamily of serine threonine kinase membrane-bound receptors. Unlike the TGF-β receptor, BMPR2 requires a “type 1” co-receptor to form a heterotetrameric complex and transduce signals in response to ligands. Currently, there are 20 Bone Morphogenetic Proteins (BMPs) which are secreted as homo- or heterodimers and signal via type 1 and type 2 receptor complexes. There are four type 1 receptors: Activin receptor-like kinase 1(ALK1), BMPR-1A (ALK3), BMPR-1B (ALK6), and ActR-1A (ALK2). There are two relevant type 2 receptors: BMPR2 (ActRIIA) and ActRIIB. The combinations of receptors vary across cell type, and confer specificity to given BMP ligands. Notably, ALK1 is generally expressed on endothelial cells but not on smooth muscle cells. Once a heterotetrameric complex is formed in response to a ligand, the type 2 receptor phosphorylates the type 1 receptor which in turn phosphorylates both canonical and noncanonical signal transduction targets. In the canonical pathway, a receptor “mothers against decapentaplegic” (R-SMAD) protein—typically SMAD 1/5/8, reviewed in [10]—is phosphorylated, complexes with a co-SMAD (typically SMAD4), enters the nucleus, binds to a BMP response element DNA sequence (BRE), and promotes expression of transcription factors such as Inhibitor of DNA Binding 1, 2, and 3 (ID1, ID2, ID3) which have critical consequences for cell migration, proliferation, and apoptosis [11]. As discussed below, BMPR2 is also able to activate noncanonical pathways, such as the Extracellular Signal-Regulated Kinase (ERK), p38 Mitogen-Activated Protein Kinase (MAPK), Lin11, Isl-1, and Mec-3 domain kinase (LIMK), NOTCH, and the Wingless (Wnt) pathway (Figure 1). The pathway is complexly regulated at multiple levels by auxiliary co-receptors (Endoglin), pseudoreceptors (BMP and Activin Membrane-Bound Inhibitor, BAMBI), BMP antagonists (Noggin, Gremlin-1, Chordin), and inhibitory SMAD proteins (SMAD6, 7), reviewed in [5]. Notably, SMAD6 is also upregulated by phospho-SMAD 1/5/8, a means for negative feedback [12]. 

Importantly, several of the additional mutations associated with adult-onset PAH involve key members of the canonical BMPR2 signaling pathway, including SMAD8 [13], Caveolin-1 [14], growth differentiation factor (GDF)2/BMP9 [15], and ALK1 [16]. Further underscoring the importance of BMP signaling in vascular homeostasis, mutations in ALK1, GDF2/BMP9, SMAD4, and Endoglin are responsible for the development of hereditary hemorrhagic telangiectasia (HHT), a disease characterized by the formation of arteriovenous malformations throughout the lung, brain, liver, skin, and mucus membranes. Interestingly, HHT and PAH can coexist in the same patient [17,18].

Notably, the penetrance of BMPR2 mutations is 20% [19], suggesting that additional genetic or environmental factors must collude in order to realize a disease phenotype, potentially by reducing BMPR2 signaling below a critical threshold. Of note, beyond heterozygous BMPR2 germ line mutations in familial PAH, both idiopathic PAH and associated PAH (APAH) due to interstitial lung disease, connective tissue disease, or congenital heart disease are associated with lower levels of BMPR2 expression and signaling [20,21]. This suggests that dysfunctional BMPR2 signaling is a key feature of PAH in general. Furthermore, blood outgrowth endothelial cells extracted from PAH patients’ peripheral blood mononuclear cells exhibit greater than 50% reduction of BMPR2 protein regardless of BMPR2 mutation status [22]. 

In PAH, a disease with multiple cell types involved, signaling pathways implicated, and temporal dynamics at play, the strong genetic association with BMPR2 creates a key opportunity for developing and testing therapeutic interventions. Therapies with genetically supported targets are more than twice as likely to make their way through the development pipeline to preclinical trials [23]. It is not surprising that there is a concerted effort to augment or rescue the BMPR2 pathway for therapy. The first demonstration of BMPR2 gene therapy was conducted successfully in 2007 in the chronic hypoxia rat model of pulmonary hypertension (PH), yet the clinical translation of a gene therapy approach is challenging [24]. Treatment with the repurposed drug FK506 (Tacrolimus), recombinant BMP9, and Elafin shows promise in animal models with clinical trials planned or currently underway [25,26,27]. Even established PAH therapies such as prostacyclins and sildenafil appear to potentiate the BMPR2 pathway somewhat, yet a direct comparison of their ability to increase BMP signaling has not been made [28,29]. A comprehensive review of BMPR2 targeted therapies is covered by Orriols et al. [30].

In PAH, the notion of universally impaired in vivo BMPR2 signaling appears to be reliably supported [20,22,31], but several questions remained unanswered. It is not known how BMPR2 becomes impaired in absence of a mutation. The aforementioned low disease penetrance for a BMPR2 mutation suggests either alterations in “BMPR2 modifier” genes that enhance genetic predisposition, or the need for a secondary insult acting on a primed substrate. 

Much insight into the BMPR2 pathway is derived from in vitro experiments studying cells in isolation. As multiple cell types share the same signaling pathway, this may not recapitulate the spatially, and likely temporally, heterogeneous in vivo signaling network. The spatiotemporal dynamics of BMPR2 ligands, type 1 receptors, and type 2 receptors in the lung may be extremely relevant to the evolution of cardiopulmonary disease.

Finally, impaired BMPR2 signaling is best studied in endothelial and smooth muscle cells and is closely linked to vascular dysfunction, yet it is known that BMPR2 is also expressed in the adult bone marrow, heart, brain, placenta, liver, skeletal muscle, pancreas, kidney, placenta, gonads, and small intestine [32]. Global understanding of BMPR2 function may allow us to understand how other organs (e.g., the immune system or the right ventricle) contribute to PAH pathogenesis. 

**Figure 2:**
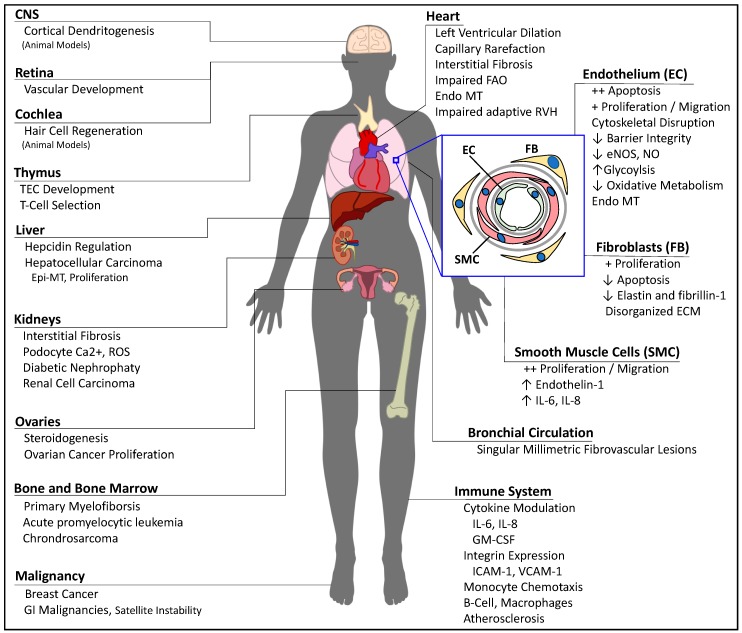
Manifestations of BMPR2 deficiency across different organ systems. CNS = central nervous system; ECM = extracellular matrix; Endo-MT = endothelial-to-mesenchymal transition; eNOS = nitric oxide synthase 3; Epi-MT = epithelial-to-mesenchymal transition; FAO = fatty acid oxidation; GI = gastrointestinal; GM-CSF = granulocyte-macrophage colony-stimulating factor; ICAM = intercellular adhesion molecule; NO = nitric oxide; ROS = reactive oxygen species; RVH = right ventricular hypertrophy; TEC = thymic epithelial cells; VCAM = vascular cell adhesion protein.

In the sections below, we define BMPR2 deficiency as either an inactivating mutation of the receptor, or an impairment of the signaling pathway, and we will examine the consequences of BMPR2 deficiency across different cellular compartments and organ systems (Figure 2).

## 2. Mechanisms for BMPR2 Deficiency

**Figure 3:**
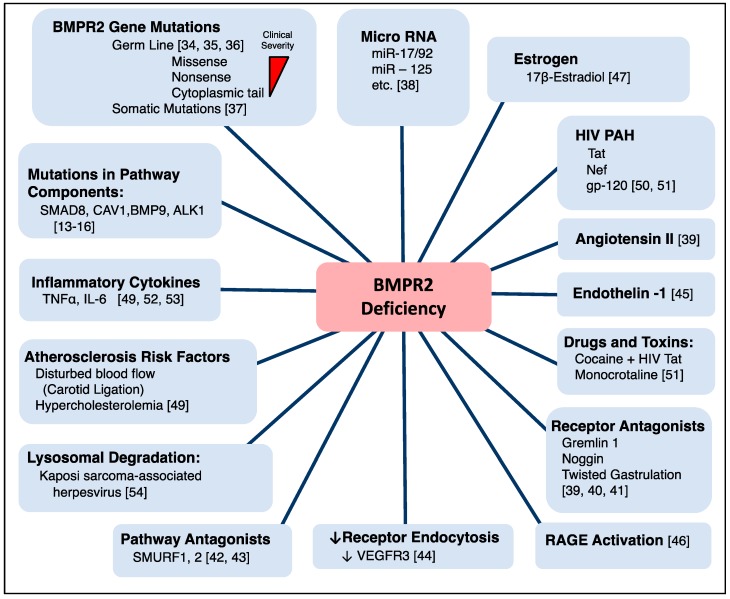
Mechanisms for BMPR2 deficiency. ALK = activin-like receptor; BMP = bone morphogenetic protein; BMPR2 = bone morphogenetic protein receptor 2; CAV1 = caveolin 1; gp-120 = envelope glycoprotein GP120; HIV = human immunodeficiency virus; Nef = negative factor; RAGE = receptor for advanced glycation end products; SMAD = mothers against decapentaplegic; SMURF = SMAD-specific E3 ubiquitin protein ligase; Tat = trans-activator of transcription; VEGFR3 = Vascular endothelial growth factor receptor 3.

There are multiple causes for downregulation of BMPR2 signaling that do not involve genetic mutations, and could contribute to reducing BMPR2 below a critical threshold needed to initiate disease (Figure 3). 

The prevalence of BMPR2 mutations in a large cohort of patients with idiopathic, heritable, and anorexigen-induced PAH was 29% [33]. As of 2017 there are 370 identified mutations in BMPR2 associated with pulmonary hypertension. Nonsense, missense, frameshift mutations, and major gene rearrangements are the most common [34]. Notably, patients with missense (versus truncating) mutations were younger at time of diagnosis or death and had shorter survival from diagnosis to death or lung transplantation [35]. This is thought to be due to a dominant negative effect on downstream BMPR2 signaling caused by stable missense mRNA transcripts, whereas transcripts containing nonsense mutations or other premature translation stop signals undergo nonsense-mediated RNA decay. However, missense mutations in the cytoplasmic tail appear to be less severe than other BMPR2 mutations with a later age of onset, lower pulmonary vascular resistance, and more vasoreactivity [36]. Additionally, second-hit somatic mutations affecting the BMPR2 pathway have been described in PAH [37]. Finally, BMPR2 deficiency can arise from mutations of members of the upstream or downstream signaling pathway, such as SMAD8, CAV1, BMP9, or ALK1.

Micro-RNA (miR) regulation of BMPR2 signaling occurs in complicated networks at multiple levels of the signaling pathway, reviewed in [38]. These miR can downregulate BMPR2 mRNA transcription under a diverse set of circumstances, such as hypoxia (miR-21, miR-125a), interleukin (IL)-6 stimulation (miR-17/92), and Human Immunodeficiency Virus (HIV) (miR-125, 301a).

BMP access to receptors is altered by numerous antagonists and the extracellular matrix, reviewed in [39,40]. Interestingly, it was shown with induced pluripotent-stem-cell-derived endothelial cells that unaffected BMPR2 mutant carriers compensate for their mutation by downregulating receptor antagonists and increasing receptor activators [41]. Downstream signaling regulators SMURF1 and SMURF2 target SMAD1 for ubiquitin degradation [42,43]. VEGFR3 can complex with BMPR2. When VEGFR3 is silenced by siRNA knockout, ligand-activated endocytosis of the BMPR2 receptor is prevented, arresting signaling [44]. This could imply a concurrent role for VEGF antagonists in inhibiting BMPR2 signaling. Endothelin-1 upregulates the antagonist gremlin 1, decreasing BMPR2 signaling [45]. Receptor for advanced glycation end products (RAGE) pathway activation reduces BMPR2 expression [46]. Finally, it is known that female lymphocytes have 50% less BMPR2 expression than their male counterparts. In fact, in pulmonary artery endothelial cells and lymphocytes, treating with 17-β estradiol causes the estrogen receptor to target a binding site in the BMPR2 gene promoter, inhibiting BMPR2 transcription [47].

There is evidence for genetic regulation of BMPR2 by “BMPR2 modifier genes”. We have identified fragile histadine triad (*FHIT*) as one such gene, which, when targeted by the drug Enzastaurin, ameliorates PAH in animal models [48]. 

Atherosclerosis risk factors, including disturbed blood flow fluid dynamics (carotid ligation), hypercholesterolemia, and angiotensin II infusion, also decrease systemic vascular expression of BMPR2 [49].

Study of HIV-associated PAH reveals that viral protein products Tat, Nef, and gp-120 reduce BMPR2 expression [50]. This effect was most dramatic in a combined HIV- and cocaine-mediated PAH model, and it implicates miR-126 and 301a as mechanisms of BMPR2 downregulation [51]. 

Inflammatory cytokines such as IL-6 and tumor necrosis factor alpha (TNF-α) can decrease BMPR2 expression via miR and by metalloprotease-mediated cleavage of the receptor [49,52,53]. Finally, BMPR2 can be prematurely ubiquitinated and degraded in lysosomes, a process which has shown to be mediated by Kaposi sarcoma-associated herpesvirus (KSHV) [54].

## 3. BMPR2 Deficiency in the Pulmonary Vasculature

Since its linkage to PAH, BMPR2 deficiency has been inexorably connected to pathologic pulmonary vascular changes and worse clinical outcomes. The seminal work by Atkinson et al. in 2002 demonstrated that relative to healthy controls, BMPR2 protein was significantly decreased in the vasculature of patients with both primary (idiopathic) and familial PAH harboring a BMPR2 mutation. Importantly, those patients with secondary PAH due to either ventricular septal defect, chronic thromboembolism, or scleroderma also had a significant decrease in vascular BMPR2 [20]. A comprehensive review of PAH pathology in patients on treatment was conducted in 2012 and found that BMPR2 mutations were associated with increased vascular intimal thickness [55]. Building on this, BMPR2 mutant PAH patients present on average 10 years earlier than non-carriers; have roughly 35% higher pulmonary vascular resistance, an 8 mmHg higher mean pulmonary artery pressure, and a 15% lower cardiac output; and are generally not vasoreactive [56,57]. Moreover, this younger BMPR2 mutant cohort was found to have a higher risk of death or lung transplantation and all-cause mortality with respect to non-BMPR2-mutant PAH patients [33]. Therefore, not only is BMPR2 vascular deficiency immediately relevant to clinical outcome but as seen below it recapitulates in vitro the multiple hallmark vascular pathologic changes seen in PAH. 

### 3.1. Consequences of BMPR2 Deficiency in the Intimal Layer: Endothelial Cells

In 1974, Meyrick et al. performed an open lung biopsy of a man with primary pulmonary hypertension and found thickening of the non-muscularized vessel endothelium by electron microscopy with luminal occlusion [58]. Pioneering research in the 1980s showed that endothelial derangements were the first pathologic change seen in patients with associated PAH due to congenital heart disease [59]. Today, endothelial cell apoptosis is generally recognized as a sentinel event in PAH evolution with a proliferation of apoptosis-resistant endothelial cells occurring later in the disease [60]. BMPR2 deficiency in endothelial cells can recreate these hallmark pathologic findings.

BMPR2 was first implicated in endothelial cell biology in 2001, when bovine aortic endothelial cells grown in tube-forming conditions on a type 1 collagen matrix upregulated BMPR2 mRNA and protein [61]. Teichert-Kuliszewska and Stewart then demonstrated that pulmonary artery endothelial cell (PAEC) apoptosis, generated by either serum-free growth conditions or TNF-α exposure, was ameliorated by treating with either BMP2 or 7, an effect entirely dependent on the BMPR2 receptor. They also showed a twofold increase in apoptosis with siRNA knockout of BMPR2 alone [62]. Interestingly, siRNA knockout of BMPR2 alone in cell culture upregulates the Ras/Raf ERK1/2 pathway, increases cell proliferation and migration, and disrupts stress fibers and focal cell adhesions [63]. These data show that in cell culture, siRNA knockout of BMPR2 alone can achieve two contradictory phenotypes—both apoptosis and proliferation. These and other studies demonstrate that through decreased barrier resilience, cell proliferation might be promoted by an increased exposure of the sub-endothelial cells to growth factors and chemokines [64]. However, the mechanism by which BMPR2 deficiency potentiates apoptosis or proliferation in vivo remains elusive. 

Further complicating the picture, multiple pathways beyond canonical SMAD signaling are influenced by BMPR2. BMP2 ligand can increase BMPR2-mediated phosphorylation of ERK, which then inactivates Glycogen Synthase Kinase 3-β (GSK3-β) degradation of β-Catenin. This ultimately facilitates the canonical Wingless (Wnt) signaling pathway and increases PAEC proliferation. Noncanonical activation of RhoA and Rac1 is facilitated by either by a SMAD1 or SMAD3 recruitment of Disheveled (Dvl) protein, which increases cell motility [65]. 

The loss of BMPR2 has direct consequences on the secretion of vasodilatory and inflammatory cytokines by endothelial cells. BMP9 signals through BMRP2 to induce the transcription of Id1, Id2, E-Selectin, IL-6, and IL-8 [66]. In PAECs subject to TNF stimulation, decreased BMPR2 (by siRNA) results in a prolonged activation of p38-MAPK2 with increased granulocyte-macrophage colony-stimulating factor (GM-CSF) mRNA translation [67]. The critical vasodilator nitric oxide (NO) is produced in the endothelium by phosphorylated endothelial nitric oxide synthase (eNOS). Not only does BMPR2 immunoprecipitate with eNOS, but there is compromised generation of phospho-eNOS after treatment with BMP2/7 when BMPR2 is deficient, either by siRNA knockdown in normal cells or study of BMPR2 mutant PAECs [68].

Endothelial cells from PAH patients exhibit a significant decrease in mitochondrial oxidative phosphorylation with an increase in aerobic glycolysis, similar to malignancies. BMPR2 deficiency in endothelial cells can potentiate these alterations in cell metabolism. Loss of BMPR2 directly induced mitochondrial dysfunction and facilitated a transition from glucose oxidation to glycolysis [69]. BMPR deficiency can potentiate an alternative splicing of pyruvate kinase, with a similar consequence of enhanced glycolysis and decreased mitochondrial oxidative phosphorylation [70].

Similar to what is observed in the lesions of PAH patients, there is increased DNA damage and impaired DNA repair which can contribute to somatic genomic mutations and microsatellite instability, potentially leading to an apoptosis-resistant phenotype [71]. Silencing of BMPR2 in endothelial cells led to increased levels of HMGA1, SNAI1, and Slug, with increased expression of Acta2, SM22α, and phospho-vimentin [72]. This so-called endothelial-to-mesenchymal transition (EndoMT) could explain the appearance of neointimal occlusive lesions that express both endothelial and smooth muscle markers.

All effects taken together, BMPR2 deficiency in endothelial cells leads to a disrupted cytoskeletal and adhesion structure, increased apoptosis, increased migration, increased production of inflammatory cytokines and vasoconstrictors, and a loss of endothelial cell identity, all of which occur in PAH. 

### 3.2. BMPR2 Deficiency in the Medial Layer: Smooth Muscle Cells

A hallmark of pathologic vascular lesions in PAH is the proliferation of smooth muscle cells (SMCs) resulting in medial hypertrophy along with the appearance of cells expressing smooth muscle cell markers in the neo-intima. Dysregulation of BMPR2 signaling is directly tied to extracellular and intracellular signaling changes which increase pulmonary artery smooth muscle cell (PASMC) migration, proliferation, and cytokine release.

Work with PASMCs has shown that active canonical BMPR2 signaling is critical for controlling the two main pathologic behaviors of PASMCs in PAH: increased proliferation and decreased apoptosis. These cells express a multitude of type 1 and type 2 BMP receptors, with BMP7 long established to decrease both PASMC proliferation and apoptosis [73,74]. Although treatment with TGF-β and multiple BMPs can quell PASMC proliferation, Morrell and colleagues first noted that PASMCs from primary (idiopathic) PAH patients were resistant to the anti-proliferative effects of BMP2, BMP4, or BMP7 versus healthy controls [75]. They subsequently showed that the anti-proliferative effect was mediated by BMPR2 canonical SMAD signaling [76]. Beyond quelling cell proliferation, BMP4, signaling through BMPR2 and SMAD 1/5/8, also drives PASMC apoptosis [77]. Although BMP signaling can occur in the absence of BMPR2 via a type 1 receptor, it was shown in PASMCs that BMPR2 is necessary and sufficient to generate the sustained signal required for intracellular accumulation of ID1 protein [78]. This was investigated further by demonstrating that both BMP4 and BMP6 require BMPR2 to trigger ID1 and ID3 expression, halt cell cycle advancement, and ameliorate PASMC proliferation [79]. BMPR2 deficiency allows unchecked proliferation of PASMCs in response to growth factors and cytokines such as platelet-derived growth factor-BB (PDGF-BB), fibroblast growth factor (FGF), and TGF-β1 [73,80]. Under normal conditions, TGF-β incompletely represses BMP4’s ability to activate ID1 and ID2. BMPR2 deficiency will allow TGF-β to operate in a completely unchecked fashion, completely repressing ID1 and ID2 transcription regardless of BMP4 signaling [81]. 

BMPR2 deficiency in muscle can potentiate proliferative paracrine signaling. Loss of BMPR2 will block the ability of BMP4 and BMP7 to repress the secretion of endothelin-1 in PASMCs. Additionally, since BMP7 antagonizes the arterial contraction caused by endothelin-1, loss of BMPR2 can generate increased endothelin-1-mediated vasoconstriction [82]. Finally, endothelin-1 itself downregulates BMPR2 expression in PASMCs, and can thereby create a positive feedback loop of vasoconstriction [45].

Similar to what is seen in PAECs, BMPR2 exists at an intersection between multiple signaling pathways in PASMCs. BMPR2 can create an imbalance in these pathways, giving rise to a proliferative and migratory phenotype. The Wnt signaling pathway is, again, one example. In smooth muscle, BMP2 signals through BMPR2 to phosphorylate Akt, suppress the degradation of β-Catenin, and increase fibronectin expression. Fibronectin incorporates into the extracellular matrix, promoting motility, growth, and contraction of smooth muscle cells [83]. Beyond Wnt signaling, BMP2 can signal through BMPR2 in PASMCs to activate PPARγ and apolipoprotein E (ApoE) transcription and decrease PASMC proliferation [84]. Additionally, the “metastasis” factor, S100 calcium binding protein A4 (S100A4), controls PASMC migration via RAGE, but does so in a manner which is co-dependent on BMPR2 [85]. Notably, RAGE activation downregulates BMPR2 expression. RAGE inhibition rescues BMPR2 expression and is being considered as a therapeutic target in PAH [46]. 

BMP 2, 4, and 7 can suppress proto-oncogene Src family protein tyrosine kinase phosphorylation through interaction with the c-terminus of BMPR2 [86]. Src kinases have multiple cellular effects including vasoconstriction and cellular proliferation. Recently, TNF-α was shown to upregulate A Disintegrin and Metalloprotease (ADAM) 10 and 17, causing cleavage of BMPR2 and shedding of its ectodomain as an extracellular ligand trap. The absence of BMPR2 allowed for sustained BMP6-mediated signaling via ActR-IIA/ALK2 and sustained Src phosphorylation. Src activation led to upregulation of NOTCH expression and increased cell proliferation [53].

Thus, BMPR2 deficiency can achieve the phenotype of increased PASMC migration and proliferation through multiple complex signaling pathways. Yet unknown is how these downstream pathways may change temporally in early versus end-stage disease or how neighboring cell types fully influence these pathways. 

### 3.3. The Tunica Adventitia and BMPR2 Deficiency: Fibroblasts and Extracellular Matrix

The adventitial layer in PAH is deranged by the infiltration of inflammatory cells, increased populations of resident and circulating fibroblasts, activated dendritic cells, overexpression of chemokines, and collagen matrix deposition [87]. Adventitial fibroblasts are the most prominent cell type, and in vascular disease they proliferate and differentiate into myofibroblasts, contributing to extracellular matrix production. The role of BMPR2 deficiency in fibroblasts supports the PAH phenotype but is not as well characterized as intimal and medial cells. 

Fibroblast proliferation and apoptosis are influenced by BMPR2. Human fetal lung fibroblasts are known to express BMP2, BMP4, and BMP7. Further, treatment of fetal lung fibroblasts with BMP4 activates canonical and noncanonical BMPR2 pathways, prevents serum-stimulated proliferation, and increases expression of the SMC maker alpha smooth muscle actin [88]. In response to BMP2 or growth differentiation factor 5 (GDF5, also known as BMP14), BMPR2 complexes with X-Linked Inhibitor of Apoptosis (XIAP) to prevent its ubiquitination and subvert apoptosis in mouse embryonic fibroblasts [89].

Disruption of the elastic lamina due to expression of neutrophil elastase is a prominent feature in PAH that allows for dissociation of matrix-bound growth and inflammatory factors. Notably, silencing of BMPR2 in pulmonary adventitial fibroblasts results in decreased production of elastin and fibrillin-1 in response to TGF-β1. Additionally, fibroblasts from BMPR2 mutant PAH patients have severely impaired elastic fiber formation [90]. Taken together, this suggests that BMPR2 deficiency leads to elastic lamina degradation, enables cellular migration, and enhances growth factor transit between cellular compartments.

Further association is demonstrated by a mouse model for scleroderma lung and skin disease. Here, a kinase-deficient TGF-β1 receptor is overexpressed in fibroblasts using the pro-α2 (I) collagen gene as a promoter. This model counterintuitively results in an overactivation of TGF-β1 target genes within fibroblasts (rather than a dominant-negative suppression of TGF-β1 signaling) and is refractory to exogenous TGF-β [91]. These mice develop dermal and pulmonary fibrosis. Treatment with the VEGF receptor inhibitor Sugen-5416 causes endothelial apoptosis, occlusive vascular lesions, and pulmonary hypertension. Notably, there is profoundly reduced expression of BMPR2 both in whole lung lysate and in explanted fibroblasts in this mouse model [92]. Although providing an intriguing association between BMPR2 deficiency and fibroblast activation, this does not imply causality between one and the other.

Fibroblasts exist in diverse subpopulations, orchestrate the production of the extracellular matrix of connective tissue, and play integral roles in mediating wound healing and inflammation, reviewed in [93]. Although there is traction for BMPR2 deficiency supporting proliferation of adventitial fibroblasts with disrupted extracellular matrix production, it is yet unexplored if BMP signaling alters the distribution of fibroblast subpopulations or the growth factors and cytokines produced by them. Although complicated by the selection of a fibroblast-specific promoter, a fibroblast-specific BMPR2 knockout animal model could provide further insight into the role of the adventitia in pulmonary hypertension.

## 4. BMPR2 Deficiency in the Systemic Circulation

Beyond what is known with HHT, descriptions of other morphologic abnormalities of the systemic vasculature in patients harboring BMPR2 mutations are sparse. BMP signaling, however, is tied to the pathogenesis of atherosclerosis, which will be covered when discussing the immune system. One exception to this is the bronchial arteries. In idiopathic PAH, the bronchial arteries are known to form communications with both the pulmonary circulation and the vaso vasorum [94]. Although bronchial arterial hypertrophy with abnormal bronchial-to-pulmonary anastomoses has been observed in chronic thromboembolic pulmonary hypertension (CTEPH), BMPR2 mutations appear to generate unique singular millimetric fibrovascular lesions (SiMFis) affecting larger bronchial arteries of 1–2 mm in diameter [95]. BMPR2 mutant patients also have an increased frequency of anastomoses between bronchial arteries, pulmonary arteries, the alveolar capillary bed, and pulmonary veins. The resultant increase in communication between systemic and pulmonary circulations could explain why higher amounts of hemoptysis are seen in PAH patients with BMPR2 mutations [33].

## 5. BMPR2 Deficiency in the Heart

Right ventricular (RV) failure is the final common pathway for end-stage PAH. Both survival and RV hemodynamics, specifically, pulmonary capacitance and right ventricular stroke work index, are worse in familial versus idiopathic PAH [96]. It was previously thought that this fact was a consequence of higher pulmonary vascular resistance, but more rigorous investigation has shown that BMPR2 mutations have additional direct pathologic influence on the heart. Patients with BMPR2 mutations have lower stroke volume index, cardiac index, and right ventricular ejection fraction relative to a matched, non-BMPR2 mutant cohort with similar RV afterloads [97]. 

Study of left ventricular heart failure in a transverse aortic constriction mouse model shows a strong interdependence between non-BMP TGF-β signaling, ventricular dilation, interstitial fibrosis, and a reduced capillary density. This was associated with noncanonical activation of transforming growth factor beta-activated kinase 1 (TAK1) by TGFβR2 [98]. BMP2 was found to subvert serum-starvation-induced apoptosis in fetal rat cardiomyocytes in a SMAD1-dependent manner. Although BMPR2 was present in these cells, it was not conclusively implicated in the activation of SMAD1 [99]. Wu et al., however, found that in the left ventricular free walls of patients with either ischemic and non-ischemic cardiomyopathy undergoing heart or heart–lung transplantation, BMPR2 expression at a protein level was consistently downregulated relative to donor hearts [100].

Currently, the effect of BMPR2 deficiency on RV cardiomyocytes is primary studied with respect to metabolism. PAH can be characterized by a mitochondrial impairment that shifts metabolism away from glucose and fatty acid oxidation [101]. It has been shown that cardiomyocytes from the failing right ventricles of PAH patients accumulate fatty acid, suggesting impaired fatty acid utilization. The resultant metabolic impairment in cardiomyocytes could explain the observation of RV dysfunction out of proportion to pulmonary vascular occlusion. Study of the BMPR2R899X mouse model by Talati, West, and Hemnes has shown that overexpression of a dominant negative mutant BMPR2 allele in cardiomyocytes led to a significant increase in deposition of long-chain fatty acids [102]. These mice do not develop RVH in response to RV afterload [103]. Notably, this lipid accumulation was ameliorated by metformin.

Given the relationship between non-BMP TGF-β signaling and cardiac interstitial fibrosis in the left ventricle, it is compelling to ask if BMPR2 deficiency imparts a further maladaptive response in the hypertensive RV, which is the topic of active investigations. This raises the possibility that BMPR2 rescue therapies could benefit both the lung and the RV.

## 6. BMPR2 Deficiency in the Immune System

Multiple lines of evidence demonstrate that inflammation is a key component in the pathogenesis of PAH, reviewed in [104]. Two key pathologic themes seen in PAH patients are (1) increased local inflammation in vascular lesions, with infiltration of T cells, B cells, macrophages, and neutrophils, and (2) a global redistribution of inflammatory cell populations. BMPR2 has a role in regulating immune system function and can contribute to both of these features.

First, BMPR2 deficiency assists in recruitment of lymphocytes, macrophages, and neutrophils to the vessel by increasing vascular cytokine production. There is increased endothelial GM-CSF production in response to BMPR2 reduction [67]. In the smooth muscle of BMPR2^+/−^ mice or in BMPR2 mutant human cell culture, BMPR2 deficiency increases IL-6 and IL-8 mRNA and protein expression in response to lipopolysaccharide administration [105]. Further, a negative feedback loop exists whereby IL-6 activates signal transducer and activator of transcription 3 (STAT3) to increase expression of micro-RNA (miR) 17-5 and miR-20. These miR bind the 5’UTR of BMPR2 mRNA, leading to its degradation [52]. 

After being recruited by cytokines, BMPR2 deficiency can aid in the diapedesis of inflammatory cells to the sub-endothelial tissue. Vascular infiltration by inflammatory cells is mediated by arterial, venous, or lymphatic transendothelial migration, a complex process orchestrated by chemokines, integrins, and adhesins, reviewed in [106]. BMPR2 regulates this process both from endothelial and immune standpoints. BMP4 is upregulated and BMPR2 is downregulated in response to disturbed blood flow in the endothelium of early systemic and coronary atherosclerotic lesions, causing expression of integrins and increased lymphocyte transendothelial migration [107]. Knockout of BMPR2 by siRNA in human PAECs was shown to increase the TNF-α-mediated transmigration of lymphocytes and neutrophils, and (in an endothelial-specific knockout mouse model) is associated with increased endothelial permeability and albumin leakage [108]. BMPR2-specific knockout alone was shown to upregulate expression of ICAM-1 and VCAM-1 in a BMP4-independent manner promoting monocyte adhesion [49]. From the standpoint of the immune system, BMPR2 is expressed on monocytes, and silencing it impairs BMP2/4-mediated chemotaxis across the endothelium [109]. Nonselective expression of the dominant negative BMPR2 allele (BMPR2^delx4+^) in mice results in constitutive activation of bone-marrow-derived macrophages, supporting a notion that active BMPR2 signaling suppresses macrophage activation and could quell inflammation [110]. Taken together, this would suggest BMPR2 deficiency creates an environment of increased endothelial–immune communication, with increased immune access to the sub-intimal blood or lymphatic vascular structures. 

The balance of immune cell populations is altered in PAH, a fact long known but quantified recently by Marsh and colleagues. Flow cytometry defined the distribution of all cluster of differentiation-45 (CD45) positive cells in the lungs of patients with and without idiopathic PAH. In addition to a striking expansion of T cells, neutrophils, macrophages, and dendritic cells, there was a previously unknown expansion of nonclassical plasmacytoid dendritic cells and γδT cells [111]. Whether this shift arises secondary to advanced PAH or existed in some form prior to disease development is an open question. However, the importance of T cells, and, by proxy, the thymus, in PH pathogenesis is elegantly demonstrated in the athymic rat PH model. Athymic rnu/rnu rats lacking T cells and treated with a VEGFR2 inhibitor develop severe pulmonary hypertension with occlusive vascular lesions at normoxia. Immune reconstitution of exogenous regulatory T cells reverses pulmonary hypertension with a significant increase in whole-lung BMPR2 expression [112]. It is possible that BMPR2 signaling deficiency could recapitulate an athymic phenotype in humans with abnormal T cell populations.

The thymus plays a central role in T cell maturation and selection. Double negative thymocytes from the bone marrow mature to double positive (CD4^+^/CD8^+^) T cells, a process which is arrested by BMP2/4 acting on the thymocyte [113]. Blocking this signal could result in ineffective negative selection of T cells. Resulting double positive T cells then interact with thymic epithelial cells (TECs) in the cortex and medulla of the thymus and undergo positive and negative selection. This imparts both self-tolerance and the ability to recognize a diverse repertoire of non-self-antigens [114]. TEC development depends on BMP4 activation of the *Foxn1* transcription factor in embryonic development, with suboptimal expression of Foxn1 resulting in thymic hypoplasia and impaired T cell development [115]. Interestingly, BMPR2 is expressed in major histocompatibility complex (MHC)-II-positive thymic epithelium, thymic mesenchyme, and CD4^−^/CD8^−^ double negative thymocytes [116]. It is possible, though unproven, that BMPR2 deficiency could alter thymic and T cell development, increasing the potential for autoimmunity. Beyond this, there is evidence that BMPs regulate the function of dendritic cells, naïve and memory B cells, natural killer cells, and macrophages, with BMPR2 being at least expressed in macrophages and B cells, reviewed in [117]. However, the dynamics of these signaling processes in a disease such as PAH are not well characterized.

It is compelling to think that BMPR2 deficiency in the immune system could conspire with a susceptible BMPR2-deficient vascular substrate to permit inflammation and vascular remodeling. The dynamics of these potentially pathologic cell–cell interactions are largely unknown. Further investigation may include recovering BMPR2 signaling in specific immune cell types or developing therapies that reconstitute an underdeveloped portion of the immune system. 

## 7. BMPR2 Deficiency in Other Organs

As ongoing work is developing the notion of PAH as a systemic disease, it is helpful to understand how BMPR2 deficiency can manifest in other organs. 

BMPR2 appears to be important in renal development and disease. In mice, stereotypic expression of BMPR2 is seen in the developing epithelial nephron structures, tip of ureters, and tubules [6]. Notably, knockout of BMP7 inhibits overall nephrogenesis [118], BMP4 knockout impairs Wolffian duct development [119], and BMP2 knockout arrests collecting duct formation. Notably, homozygous BMP7^−/−^ mutations are lethal within 48 h of birth, possibly due to cardiac cushion defects. In adult kidney podocytes, BMPR2 is expressed at a transcript level, but not conclusively at a protein level. BMP2-mediated expression of ID1 was shown to increase podocyte intracellular calcium and reactive oxygen species production [120]. BMP7 was tried as a treatment for kidney interstitial fibrosis, showing mainly gains in glomerular filtration rate versus Enalapril [121]. In a streptozotocin rat model of diabetes, there was a reduction in kidney and mesangial cell BMPR2 and BMP7 expression, both of which were restored after giving insulin or inhibiting glucose transporters with phloridzin [122]. Renal cell carcinoma exhibits loss of BMPR2 expression, a feature that seems prominent with higher Furhman grade (a pathologic grading inversely correlating with survival). Finally, BMP6 may play a role in decreasing renal cell carcinoma proliferation [123].

BMPR2 is expressed in liver hepatocytes. Combined inhibition of both actin-related protein 2 (ActR2a) and BMPR2 suppressed hepcidin expression, leading to impaired iron metabolism and iron overload [124]. Also notable is that BMP9 expression is increased in hepatocellular carcinoma (HCC) and enhances tumor cell migration. BMP9 also upregulates SNAI1 expression in HCC cell lines, although in an ALK1-dependent manner, and induces epithelial-to-mesenchymal transition [125]. Although not a consequence of BMPR2 deficiency, these issues could be significant in certain patients treated with BMPR2 rescue therapies.

BMPR2 is expressed in human oocytes, follicles, and corpus lutea [126], and may be upregulated in response to androgen (e.g., polycystic ovarian syndrome) [127]. BMP9 can act through BMPR2 to promote ovarian cancer proliferation [128]. Finally, study of ovarian cancer cell lines indicates BMP-15-mediated BMPR2 signaling regulates steroidogenesis by decreasing production of progesterone in response to follicle stimulating hormone (FSH) stimulation [129].

In the developing murine retina, BMPR2 is highly expressed at the vascular front and essential for retinal angiogenesis [130]. This could conceivably provide a convenient means to study the effect of cell-specific BMPR2 knockout on angiogenesis. Work investigating hearing loss has revealed that BMPR2 is expressed in avian cochlear hair cells, and that BMP4 acts to antagonize their regeneration [131]. The generation of neuronal dendrites is essential for forming proper connections in the developing mouse cortex. BMPR2 is expressed in the axon and dendrites of cortical neurons, where its cytoplasmic tail binds to LIMK, activating cofilin and promoting dendritogenesis [132].

## 8. BMPR2 Deficiency in Bone Marrow

There is a high incidence of PAH in myeloproliferative disorders, generally attributed to cytotoxic chemotherapy, tumor microemboli, and bone-marrow-derived angiogenic factors [133]. Notably, relative to patients with secondary myelofibrosis (MF) or healthy controls, patients with idiopathic MF have reduced platelet and granulocyte BMPR2 mRNA with elevated mononuclear BMPR2 mRNA [134]. In primary myelofibrosis, BMPR2 is similarly downregulated in circulating CD34^+^ cells [135]. Interestingly, in late-stage MF, bone-marrow-derived fibroblasts had upregulated BMPR2 mRNA [136]. This evidence suggests that BMPs are involved in bone marrow extracellular matrix generation; however, the dynamics of this signaling in MF are not fully elucidated.

## 9. BMPR2 Deficiency in Malignancy

After the discovery that loss of BMPR1A leads to the development of Juvenile Polyposis [137], it was suggested that BMP signaling is tumor suppressive while classic TGF-β signaling is implicated in cancer pathogenesis. There is now increasing evidence that BMP signaling and BMPR2 play roles in tumor cell migration, metastasis, apoptosis, and local inflammation in a dynamic and complex manner, reviewed in [7]. For instance, breast cancer pathogenesis and BMP signaling appear to be related, with somewhat different results in human tissue versus mouse models. Owens and colleagues suppressed BMPR2 signaling in transgenic mice by overexpressing a mutated BMPR2-DelEx4-DN allele. These mice were additionally engineered to express mouse mammary tumor virus polyoma middle tumor antigen, resulting in the development of mammary tumors. BMPR2 suppression increased tumor cell proliferation, migration, and invasion in addition to creating a more inflammatory tumor microenvironment [138]. In patients, however, BMPR2 is overexpressed in breast cancer, with levels correlating to poorer relapse-free survival. Further, genes encoding members of the BMP signaling pathway were rarely mutated in breast cancer [139]. 

The expression of BMPR2 in chondrosarcoma also directly correlates to a significantly reduced relapse-free survival. BMPR2 in chondrosarcoma stabilizes XIAP, leading to apoptosis resistance. Knockout of BMPR2 in chondrosarcoma with siRNA destabilized XIAP, increased tumor apoptosis, suppressed tumor growth, and increased autophagy [140].

On the other hand, BMPR2 is expressed in ovarian cancer, with levels correlating to improved survival [141]. This could potentially be related to ligand specificity, but more data is required.

In other cases, BMP signaling may influence the severity of malignancy. In prostate cancer, BMP6 is noted to upregulate ID1 expression, increasing invasiveness [142]. Acute promyelocytic leukemia bone marrow expresses BMPR2 and has high BMP expression levels which resolve after treatment with all-*trans* retinoic acid [143]. 

BMPR2 abnormalities are also described in GI malignancy. Somatic frameshift mutations in gastric and colorectal cancers are associated with microsatellite instability and increased epithelial growth and polyp formation [144,145]. 

Finally, BMP signaling is important in bladder cancer. The progression of urothelial cancer from carcinoma in situ to invasive carcinoma is mediated by a loss of sonic hedgehog (Shh) signaling from the bladder epithelium to the stroma. Loss of Shh from de-differentiated bladder epithelium decreases stromal BMP4/5 production. Treatment with FK506 replenished the BMP signal from stromal cells and induced differentiation of pre-malignant cells to normal urothelial cells, preventing invasive carcinoma [146].

## 10. Conclusions

BMPR2 deficiency is clearly central to the pathogenesis of PAH as it both is associated with a worse clinical outcome and recapitulates many of the cellular pathologic features of PAH. Despite significant and detailed knowledge of the receptor and its pathway, there are many questions that remained unanswered.

Many factors downregulate BMPR2 signaling and receptor expression outside of germ line and somatic mutations. As BMPR2 haploinsufficiency in patients alone is not sufficient to alter pulmonary vascular resistance or RV functional reserve [147], what genes or factors either provide the second hit, or reduce BMPR2 signaling to the level required to become pathogenic? As outlined, a BMPR2-deficient immune system may have the potential to develop auto-reactivity and act as the second hit. Additionally, we are just now beginning to understand the genetic underpinnings of BMPR2 transcriptional regulation. Elucidating BMPR2 potentiating modifier genes such as *FHIT* can reveal novel pharmacologic therapies [48]. 

Growing evidence suggests that PAH is a disease with systemic manifestations. Metabolic derangements and insulin resistance appear to be the most clinically significant pathologic manifestation outside of the heart and lungs, with growing support for pathologic renal involvement. However, as reviewed, there is potential for BMPR2 deficiency to alter immune competency, generate RV fibrosis, and cause RV capillary rarefaction. Beyond this, understanding BMPR2 signaling in tissues outside the lung may allow us to predict whether there could be untoward effects from BMPR2 modulatory therapy or if specific levels of BMP signaling are needed to maintain homeostasis in certain tissues. As noted above, BMP activation in certain cancers such breast cancer, hepatocellular carcinoma, or chondrosarcoma may be pathologic.

Still the question remains as to how BMPR2 deficiency results in dramatic plexiform lesions in a relatively specific region of a single organ. It is known that the smooth muscle proliferative response to BMP4 varies in proximal versus distal PASMCs [76]. It may also be possible that specific intimal or adventitial cell subpopulations, which can now be characterized through single-cell RNA sequencing, are responsible for this geographic phenomenon. Still open is the question of how local expression of BMP ligands is changed in disease versus health. Spatial in situ profiling of this could provide insight into the signaling dynamics within plexiform lesions. Notably, a population of T cells undergo tissue-selective T cell imprinting and home to the lung [148]. It would be interesting if BMPR2 deficiency could derange this trafficking system, either through alterations in endothelial ligand expression, or by altering T cell specificity.

Our current, detailed knowledge of BMPR2 signaling is extrapolated from cell culture from patient cell lines, study of normal cell lines, and animal models. The BMPR2 receptor can be thought of as the tip of an iceberg over a sea of complex interwoven signaling networks. Cellular crosstalk may be extremely important, and it is not clear that cell culture experiments recapitulate the in vivo signaling dynamics between endothelium, smooth muscle cells, adventitia, and immune cells. Does the robust BMPR2 protein immunostaining in the endothelium [20,149] imply that the endothelium is the main causative cell type in disease? Perhaps co-culture systems or in vivo, in situ transcriptome profiling of the downstream signaling could better delineate how a given cell type is implicated in the disease pathogenesis. Data such as this could guide the development of cell-specific therapies.

Our understanding of BMPR2’s role in PAH pathogenesis spans 20 years and highlights the BMPR2 pathway as a current focus of pulmonary vascular research. Understanding the many pathologic consequences of BMPR2 deficiency in other organ systems and the interaction with the pulmonary vasculature is key to fully understanding and targeting the BMPR2 pathway in PAH.

## Abbreviations

ActR	Activin receptor
Akt	Protein kinase b
ALK	Activin-like receptor
ApoE	Apolipoprotein E
BAMBI	BMP and activin membrane-bound inhibitor
BMP	Bone morphogenetic protein
BMPR2	Bone morphogenetic protein receptor type 2
c-Src	Proto-oncogene tyrosine-protein kinase Src
Cav1	Caveolin 1
CLIC4	Chloride intracellular channel 4
Dvl	Disheveled
eNOS	Nitric oxide synthase 3
ERK	Extracellular signal-regulated kinase
FGF	Fibroblast Growth Factor
GM-CSF	Granulocyte macrophage-colony stimulating factor
gp-120	Envelope glycoprotein GP120
GSK3-β	Glycogen synthase kinase 3-β
GDF	Growth differentiation factor
HIV	Human immunodeficiency virus
ICAM	Intracellular adhesion molecule
ID	Inhibitor of differentiation
IL	Interleukin
JNK	c-Jun N-terminal kinase
LIMK	Lin11, Isl-1, and Mec-3 domain kinase
MAPK	Mitogen-activated protein kinase
Nef	Negative factor
PAH	Pulmonary arterial hypertension
PDGF	Platelet derived growth factor
PPARγ	Peroxisome proliferator-activated receptor gamma
Rac1	Ras-related C3 botulinum toxin substrate 1
RAGE	Receptor for advanced glycation end products
RhoA	Ras homolog gene family member A
S100A4	S100 Calcium Binding Protein A4
SMAD	Mothers against decapentaplegic
SMURF	SMAD specific E3 ubiquitin protein ligase
STAT3	Signal transducer and activator of transcription 3
Tak1	TGF-β activated kinase 1
Tat	Trans-activator of transcription
TGF	Transforming Growth Factor
TNF	Tumo necrosis factor
VCAM	Vascular cell adhesion protein
VEGFR3	Vascular endothelial growth factor receptor 3
Wnt	Wingless
